# The polymorphism rs4705342 in the promoter of miR-143/145 is related to the risk of epithelial ovarian cancer and patient prognosis

**DOI:** 10.3389/fonc.2023.1122284

**Published:** 2023-04-04

**Authors:** Jian Zhao, Weiwei Zuo, Yue Zhang, Caiyun He, Wei Zhao, Tongyu Meng

**Affiliations:** ^1^ Department of Gynecology, The People’s Hospital of Shijiazhuang, Hebei Medical University, Shijiazhuang, Hebei, China; ^2^ Department of Gynecology, The People’s Hospital of Tangshan, Tangshan, Hebei, China; ^3^ Department of Gynecology, The Fourth Hospital of Hebei Medical University, Shijiazhuang, Hebei, China

**Keywords:** ovarian cancer, rs4705342, rs353292, risk, prognosis

## Abstract

**Objective:**

To evaluate the effects of two genetic variants in the promoter of the *miR-143/145* cluster on the risk of epithelial ovarian cancer (EOC) and the prognosis of EOC patients.

**Study design:**

Genotypes were determined by the polymerase chain reaction and ligase detection reaction method in 563 EOC patients and 576 healthy women. The expression of miR-143 and miR-145 were detected by quantitative real-time polymerase chain reaction (qRT–PCR) in fifty-two EOC tissues.

**Results:**

The rs4705342 CC genotype frequencies in EOC patients were higher than those in the controls (*P* = 0.014). Furthermore, the CC genotype of rs4705342 was associated with an advanced FIGO stage of EOC patients (*P* = 0.046). Patients with the rs4705342 CC genotype had shorter progression-free survival (PFS) and overall survival (OS) times than those carrying the TT genotype in multivariable analysis adjusting for clinical variables (HR = 1.30, 95% CI = 1.04-1.62, *P* = 0.020; HR = 1.33, 95% CI = 1.05-1.70, *P* = 0.020). In addition, the miR-145 levels were lower in EOC tissues with the rs4705342 CC genotype than in those with the TT genotype (*P* = 0.005).

**Conclusion:**

The CC genotype of rs4705342 was related to an increased risk of EOC and poor prognosis of EOC patients, and rs4705342 may serve as a molecular marker for predicting the development of EOC and the clinical outcome of EOC patients.

## 1 Introduction

Epithelial ovarian cancer (EOC) is a common malignancy of the female reproductive tract and remains the most lethal gynecologic cancer in China (1). Due to the rarity of obvious symptoms, most EOC patients are diagnosed in late stages. Currently, the main management for advanced-stage EOC is primary cytoreductive surgery, supplemented by platinum-based chemotherapy (2). Although many EOC patients show a good response to the initial therapy, most patients with advanced EOC suffer from relapse within the first 2 years. The 5-year survival rate remains approximately 40% for advanced EOC (3). Late diagnosis is one of the chief causes of the poor prognosis. Therefore, it is essential to identify potential molecular markers that could help to predict the risk of EOC.

MicroRNAs (miRNAs) are a group of small, noncoding RNAs that play an important role in modulating gene expression by targeting mRNA transcripts for their degradation or inhibiting their translation (4). Growing evidence has confirmed that miRNAs mediate multiple biological processes, including carcinogenesis (5–7). The expression of many miRNAs was found to be altered in ovarian cancer (8, 9). Of them, miR-143 and miR-145 were two common downregulated miRNAs in EOC. Previous studies have indicated that the expression levels of miR-143 and miR-145 were reduced in ovarian cancer tissues, and overexpression of the two miRNAs can inhibit EOC cell proliferation and migration, and induce EOC cell apoptosis (10–13). Moreover, EOC patients with low levels of miR-145 had a worse clinical outcome (14). These data suggested that miR-143 and miR-145 may serve as promising therapeutic targets for EOC.

It was reported that genetic polymorphisms in the promoter region of miRNAs could influence the expression of mature miRNAs and increase the risk of some cancers (15, 16). *MiR-143* and *miR-145* are two overlapping genes located on human chromosome 5q32. To date, several polymorphisms have been identified in the *miR-143/145* cluster. Among these polymorphisms, two widely studied polymorphisms (rs4705342 and rs353292) have been reported to be correlated with the risk of tumor development and cancer progression (17–20). In this study, we evaluated the effects of the two genetic variants on the risk of EOC and the prognosis of EOC patients from North China. We also detected the expression of miR-143 and miR-145 in EOC tissues with different genotypes of rs4705342 and rs353292.

## 2 Materials and methods

### 2.1 Study subjects

This study included 563 patients with EOC and 576 healthy women. All EOC patients were recruited consecutively at the Fourth Hospital of Hebei Medical University from June 2009 to December 2020. Inclusion criteria for the case group were newly diagnosed and histopathologically confirmed primary EOC. The exclusion criteria consisted of patients with other types of malignant tumors and a history of adjuvant chemotherapy or preoperative radiotherapy. Clinical and pathological information for each EOC patient was retrieved from her medical chart. The controls were cancer-free women confirmed either by ultrasound examination (n = 224), or surgical exploration (cesarean section: n = 79; hysterectomy for dysfunctional uterine bleeding: n = 273). Exclusion criteria for the control group were no personal or family history of malignant neoplasms. The control group was selected at the same period from the same hospital of case recruitment.

All EOC patients who received six to eight cycles of chemotherapy after cytoreductive surgery were followed-up from June 2009 to October 2021. Among them, 305 patients with EOC were followed-up for more than 5 years. Patients’ physical examination signs, clinical symptoms, serum CA-125 levels, and imaging examination data were collected to assess the relapse of EOC. Disease recurrence was defined as a biopsy-verifified appearance of a new lesion, and/or the appearance of new lesions on imaging examination. Progression-free survival (PFS) was defined as the time from cytoreductive surgery to the date of the documented first disease relapse or disease progression. Overall survival (OS) was defined as the time from cytoreductive surgery to the date of death from any cause.

The study was conducted with the approval of the Ethics Committee of the Fourth Hospital of Hebei Medical University. An informed consent was acquired from all participants in accordance with the Declaration of Helsinki. This study was conducted according to the Strengthening the Reporting of Observational Studies in Epidemiology criteria (21).

### 2.2 DNA extraction

Peripheral venous blood (5 mL) was drawn from all participants into vacutainer tubes containing ethylenediaminetetraacetic acid (EDTA) and then stored at 4°C. DNA was extracted from the collected blood samples by the salting-out method (22). The quality and concentration of the DNA was checked by measuring the absorbance at 260 nm and 280 nm using a UV spectrophotometer (NanoDrop 2000; Thermo Scientific, USA).

### 2.3 Genotyping

Genotyping of rs4705342 and rs353292 was performed by Shanghai Generay Biotech Co., Ltd. (http://www.generay.com.cn) utilizing polymerase chain reaction (PCR) with a ligase detection reaction method. The PCR primers for rs4705342 and rs353292 were (F) 5’-GGCTAGATGCGGCAGACC-3’ and (R) 5’-CCATGCCCCACCTTTATGC -3’. After the PCR products were subjected to ligase detection, the reaction products were analyzed using an ABI3730XL DNA sequencer (Applied Biosystems, USA). Moreover, approximately 10% of the PCR products were selected at random for repeated assays, and the results were 100% concordant.

### 2.4 RNA isolation and quantitative real-time PCR

To analyze the effect of the two polymorphisms on the gene expression of miR-143 and miR-145, fifty-two EOC tissues were collected from the abovementioned case subjects at the time of the primary cytoreductive surgery. All EOC tissue samples were stored in RNAlater solution immediately after surgical removal. Total RNA was extracted using TRIzol reagent (Generay Biotech Co., LTD, China). The quality and concentration of the extracted RNA was testeded with a UV spectrophotometer (NanoDrop 2000; Thermo Scientific, USA). The miRNAs were reverse transcribed to synthesize the first-strand cDNA with the riboSCRIPT™ Reverse Transcription Kit (RiboBio Co., Ltd., Guangzhou, China). qRT-PCR was conducted using miRNA Universal SYBR qPCR Master Mix (Vazyme Biotech Co., Ltd, Nanjing, China). The relative expression levels of miR-143 and miR-145 were obtained by the 2^-ΔΔCt^ method, and each reaction was performed in triplicate.

### 2.5 Statistical analysis

SPSS 24.0 software package (SPSS Inc., Chicago, IL, USA) was utilized for statistical analysis. Hardy-Weinberg analysis of the genotype frequencies in the control group was evaluated using the chi-square test. Genotype frequencies in the cases and controls were compared using the chi-square test with Bonferroni correction. Unconditional multiple logistic regression models were applied to estimate the odds ratio (OR) and 95% confidence interval (CI). The Kaplan-Meier method with log-rank test was used to plot the survival curves. The association of the two polymorphisms with EOC patient prognosis was assessed by univariate and multivariate survival analyses. Differences in the expression levels of miR-143 and miR-145 among different genotypes were analyzed by the Mann-Whitney U test with Bonferroni correction. *P* < 0.05 was considered significant.

## 3 Results

### 3.1 Subject characteristics

The mean age of the EOC patients was 54.28 ± 10.35 years, and the mean age of the controls was 53.29 ± 11.39 years. The age distribution was similar in the case and control groups (*P* = 0.126). Moreover, the patients and controls were also adequately matched in menstruation status (*P* = 0.124). The genotype distribution of rs4705342 and rs353292 in the controls did not deviate significantly from the expected for Hardy-Weinberg equilibrium (*P* = 0.957 and 0.971, respectively).

### 3.2 Association between rs4705342 and rs353292 polymorphisms and the risk of EOC

Genotype frequencies of rs4705342 and rs353292 in the EOC patients and controls were summarized in **Table 1**. The frequencies of rs4705342 CC genotype in the cases were significantly higher than those in the controls (*P* = 0.014). Compared with the rs4705342 TT genotype, the CC genotype was related to a higher risk of developing EOC (OR: 1.60; 95% CI = 1.10-2.34). After adjustment for age and menstruation status, the rs4705342 CC genotype was also associated with a higher risk of EOC (adjusted OR: 1.60; 95% CI = 1.10-2.33; *P* = 0.014). However, no significant difference was found in the genotype distributions of rs353292 between the two groups (**Table 1**).

**Table 1:** Association of rs4705342 and rs353292 polymorphisms with the risk of epithelial ovarian cancer.

Genotypes	Controls (%) n = 576	Cases (%) n = 563	*P*	OR (95% CI)	*P* ^a^	OR (95% CI) ^a^
rs4705342						
TT	269 (47.6)	232 (41.2)		reference		reference
TC	247 (42.9)	248 (44.0)	0.231	1.16 (0.91-1.49)	0.225	1.17 (0.91-1.50)
CC	60 (10.4)	83 (14.7)	**0.014**	1.60 (1.10-2.34)	**0.014**	1.60 (1.10-2.33)
rs353292						
GG	443 (76.9)	423 (75.1)		reference		reference
GA	125 (21.7)	129 (22.9)	0.586	1.08 (0.82-1.43)	0.597	1.08 (0.82-1.43)
AA	8 (1.4)	11 (2.0)	0.437	1.44 (0.57-3.62)	0.388	1.50 (0.60-3.78)

Data were expressed as a number (percentage), and analyzed by chi-square test.

Bold values are significant.

OR, Odds ratio; CI, Confidence interval.

a Adjusted for age and menstruation status.

P-values in bold were statistically significant.

### 3.3 Association between rs4705342 and rs353292 polymorphisms and the clinical characteristics of EOC patients

The EOC patients’ clinical characteristics were shown in **Table 2**. The correlation between the two polymorphisms and the clinicopathological parameters of the EOC patients, such as age, FIGO stage, grade, histology and residual tumor, was assessed. The results showed that rs4705342 and rs353292 were not associated with the age, grade, histology or tumor residual status of the EOC patients (**Table 2**). However, the CC genotype of rs4705342 was associated with an advanced FIGO stage of the EOC patients (*P* = 0.046), indicating that rs4705342 CC genotype might play an important role in the progression of EOC.

**Table 2:** Association of rs4705342 and rs353292 polymorphisms with the clinical characteristics of patients with epithelial ovarian cancer.

Group	rs4705342 (T/C)			*P*	rs353292 (G/A)		*P*
	TT (n, %)	TC (n, %)	CC (n, %)		GG (n, %)	GA + AA (n, %)	
Age (years)							
< 50	71 (40.1)	82 (46.3)	24 (13.6)	0.732	132 (74.6)	45 (25.4)	0.836
≥ 50	161 (41.7)	166 (43.0)	59 (15.3)		291 (75.4)	95 (24.6)	
FIGO stage							
I ~ II	74 (48.1)	65 (42.2)	15 (9.7)	**0.046**	114 (74.0)	40 (26.0)	0.709
III ~ IV	158 (38.6)	183 (44.7)	68 (16.6)		309 (75.6)	100 (24.4)	
Grade							
1 ~ 2	127 (40.7)	146 (46.8)	39 (12.5)	0.163	237 (76.0)	75 (24.0)	0.612
3	105 (41.8)	102 (40.6)	44 (17.5)		186 (74.1)	65 (25.9)	
Histology							
Serous	157 (40.6)	172 (44.4)	58 (15.0)	0.883	258 (74.4)	99 (25.6)	0.513
Endometrioid	45 (40.2)	49 (43.8)	18 (16.1)		88 (78.6)	24 (21.4)	
Mucinous	16 (47.1)	13 (38.2)	5 (14.7)		27 (79.4)	7 (20.6)	
Others	14 (46.7)	14 (46.7)	2 (6.7)		20 (66.7)	10 (33.3)	
Tumor residual							
0 cm	61 (44.2)	63 (45.7)	14 (10.1)	0.401	105 (76.1)	33 (23.9)	0.706
≤ 1 cm	59 (43.4)	55 (40.4)	22 (16.2)		105 (77.2)	31 (22.8)	
> 1 cm	112 (38.8)	130 (45.0)	47 (16.3)		213 (73.7)	76 (26.3)	

Data were expressed as a number (percentage), and analyzed by chi-square test.

Bold values are signifificant.

FIGO, International Federation of Gynecology and Obstetrics.

P-values in bold were statistically significant.

### 3.4 Association between rs4705342 and rs353292 polymorphisms and the prognosis of EOC patients

**Figure 1:**
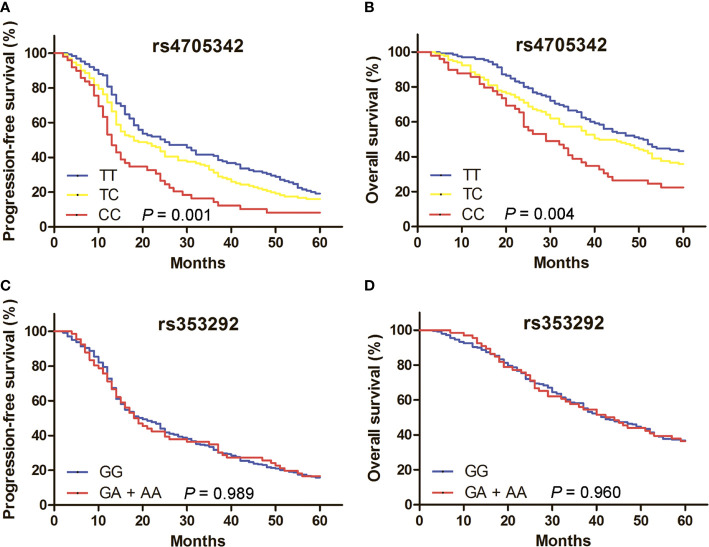
Kaplan-Meier survival curves evaluate the prognosis of 305 epithelial ovarian cancer (EOC) patients based on rs4705342 and rs353292 genotypes. **(A)** Median progression-free survival (PFS) of EOC patients carrying the TT, TC and CC genotypes of rs4705342 were 25.0, 18.0 and 13.0 months, respectively (*P* = 0.001). **(B)** Median overall survival (OS) of EOC patients carrying the TT, TC and CC genotypes of rs4705342 were 51.0, 41.0 and 29.0 months, respectively (*P* = 0.004). **(C)** Median PFS of EOC patients carrying the GG and GA + AA genotypes of rs353292 were 20.0 and 18.0 months, respectively (*P* = 0.989). **(D)** Median OS of EOC patients carrying the GG and GA + AA genotypes of rs353292 were 42.0 and 42.0 months, respectively (*P* = 0.960).

Of the 563 EOC patients, 305 were followed-up for more than 5 years. Kaplan-Meier survival analysis showed that rs4705342 was associated with the PFS and OS of EOC patients (**Figures 1A, B**; *P* = 0.001 and 0.004, respectively). Patients carrying the rs4705342 CC genotype had significantly shorter PFS and OS times than those carrying the TT genotype in a univariate Cox proportional hazards regression model (**Tables 3**, **4**; HR = 1.44, 95% CI = 1.16-1.79, *P* = 0.001; HR = 1.43, 95% CI = 1.13-1.81, *P* = 0.003). The significant correlations were maintained by multivariate analyses adjusting for age, FIGO stage, grade, histology and tumor residual (**Tables 3**, **4**; HR = 1.30, 95% CI = 1.04-1.62, *P* = 0.020; HR = 1.33, 95% CI = 1.05-1.70, *P* = 0.020). For rs353292, survival analysis demonstrated that this polymorphism was not associated with the clinical outcomes of EOC patients (**Figures 1C, D**; **Tables 3**, **4**).

**Table 3:** Association of variables with the 5-year progression-free survival of epithelial ovarian cancer patients.

Variables	n = 305	No. of recurrence (%)	Univariate model		Multivariate model	
			HR (95% CI)	*P*	HR (95% CI) ^a^	*P* ^a^
Age (years)						
< 50	99	71 (71.7)	reference		reference	
≥ 50	206	185 (89.8)	1.82 (1.38-2.40)	**<0.001**	1.43 (1.08-1.90)	**0.012**
FIGO stage						
I ~ II	79	44 (55.7)	reference		reference	
III ~ IV	226	212 (93.8)	3.62 (2.60-5.04)	**<0.001**	2.27 (1.39-3.70)	**0.001**
Grade						
1 ~ 2	169	135 (79.9)	reference		reference	
3	136	121 (89.0)	1.22 (0.96-1.56)	0.108	1.06 (0.82-1.37)	0.641
Histology						
Serous	208	184 (88.5)	reference		reference	
Mucinous	11	6 (54.5)	0.38 (0.17-0.86)	0.020	0.93 (0.39-2.18)	0.860
Endometrioid	67	51 (76.1)	0.63 (0.46-0.86)	**0.004**	0.79 (0.57-1.08)	0.142
Others	19	15 (78.9)	0.67 (0.40-1.14)	0.141	1.26 (0.72-2.19)	0.421
Tumor residual						
0 cm	65	37 (56.9)	reference		reference	
≤ 1 cm	86	70 (81.4)	1.93 (1.30-2.88)	**0.001**	1.06 (0.62-1.82)	0.834
> 1 cm	154	149 (96.8)	4.64 (3.20-6.72)	**<0.001**	2.14 (1.25-3.68)	**0.006**
rs4705342						
TT	125	101 (80.8)	reference		reference	
TC	131	110 (84.0)	0.93 (0.78-1.01)	0.369	0.95 (0.80-1.13)	0.532
CC	49	45 (91.8)	1.44 (1.16-1.79)	**0.001**	1.30 (1.04-1.62)	**0.020**
rs353292						
GG	239	201 (84.1)	reference		reference	
GA + AA	66	55 (83.3)	1.00 (0.86-1.16)	0.990	1.05 (0.90-1.23)	0.514

Data were expressed as a number (percentage), and analyzed by univariate and multivariate Cox proportional hazards models.

Bold values are signifificant.

HR, Hazard ratio; CI, Confidence interval; FIGO, International Federation of Gynecology and Obstetrics.

^a^Adjusted for age, FIGO stage, grade, histology, and residual tumor.

P-values in bold were statistically significant.

**Table 4:** Association of variables with the 5-year overall survival of epithelial ovarian cancer patients.

Variables	n = 305	No. of death (%)	Univariate model		Multivariate model	
			HR (95% CI)	*P*	HR (95% CI) ^a^	*P ^a^*
Age (years)						
< 50	99	48 (48.5)	reference		reference	
≥ 50	206	145 (70.4)	1.80 (1.30-2.20)	**<0.001**	1.45 (1.04-2.02)	**0.027**
FIGO stage						
I ~ II	79	22 (27.8)	reference		reference	
III ~ IV	226	171 (75.7)	4.53 (2.90-7.08)	**<0.001**	2.39 (1.29-4.45)	**0.006**
Grade						
1 ~ 2	169	104 (61.5)	reference		reference	
3	136	89 (65.4)	1.17 (0.88-1.55)	0.285	1.08 (0.80-1.44)	0.615
Histology						
Serous	208	145 (69.7)	reference		reference	
Mucinous	11	4 (36.4)	0.41 (0.15-1.12)	0.082	1.38 (0.48-3.92)	0.548
Endometrioid	67	33 (49.3)	0.56 (0.38-0.82)	**0.003**	0.73 (0.49-1.07)	0.107
Others	19	11 (57.9)	0.70 (0.38-1.30)	0.258	1.56 (0.82-2.97)	0.180
Tumor residual						
0 cm	65	19 (29.2)	reference		reference	
≤ 1 cm	86	44 (51.2)	2.00 (1.17-3.43)	**0.012**	1.18 (0.60-2.32)	0.637
> 1 cm	154	130 (84.4)	5.81 (3.58-9.45)	**<0.001**	2.86 (1.46-5.59)	**0.002**
rs4705342						
TT	125	71 (56.8)	reference		reference	
TC	131	84 (64.1)	0.94 (0.78-1.15)	0.558	0.92 (0.76-1.12)	0.393
CC	49	38 (77.6)	1.43 (1.13-1.81)	**0.003**	1.33 (1.05-1.70)	**0.020**
rs353292						
GG	239	151 (63.2)	reference		reference	
GA + AA	66	42 (63.6)	1.00 (0.85-1.19)	0.960	1.02 (0.86-1.22)	0.814

Data were expressed as a number (percentage), and analyzed by univariate and multivariate Cox proportional hazards models.

Bold values are significant.

HR, Hazard ratio; CI, Confidence interval; FIGO, International Federation of Gynecology and Obstetrics.

^a^Adjusted for age, FIGO stage, grade, histology, and residual tumor. P-values in bold were statistically significant.

### 3.5 Influence of rs4705342 and rs353292 polymorphisms on the expression of miR-143 and miR-145

**Figure 2:**
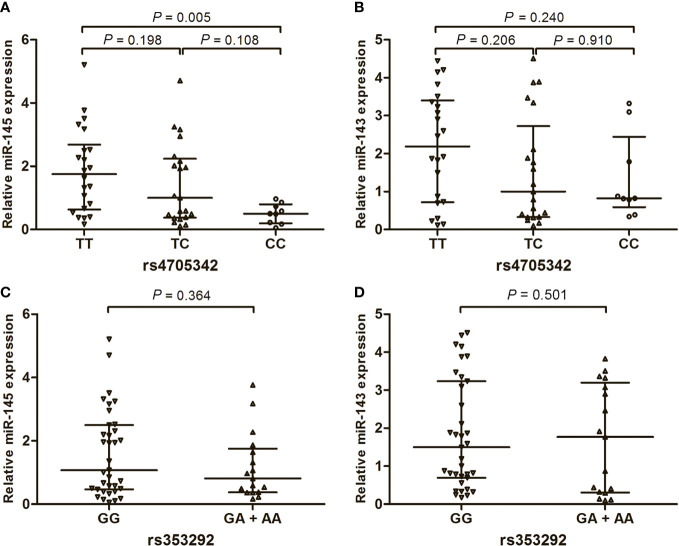
Influence of rs4705342 and rs353292 on the expression of miR-143 and miR-145 in tumor tissues from 52 epithelial ovarian cancer patients. Data were expressed as median with interquatile range, and analyzed by the Mann-Whitney U test with Bonferroni correction. **(A)** Relative expression levels of miR-145 in the ovarian cancer tissues from patients with the different genotypes of rs4705342. **(B)** Relative expression levels of miR-143 in the ovarian cancer tissues from patients with the different genotypes of rs4705342. **(C)** Relative expression levels of miR-145 in the ovarian cancer tissues from patients with the different genotypes of rs353292. **(D)** Relative expression levels of miR-143 in the ovarian cancer tissues from patients with the different genotypes of rs353292.

To explore the impact of rs4705342 and rs353292 on gene expression, we detected the expression of miR-143 and miR-145 in EOC tissues from patients with different genotypes of the two polymorphisms. The results showed that the miR-145 expression levels were significantly lower in EOC tissues from patients with the rs4705342 CC genotype than in those from patients with the TT genotype (**Figure 2A**; *P* = 0.005), indicating that rs4705342 plays a role in the regulation of miR-145 expression. However, the miR-143 expression levels were not significantly different among the different genotypes of rs4705342 (**Figure 2B**). The rs353292 polymorphism had no effect on the expression of either miR-145 or 143 in the EOC tissues (**Figures 2C, D**).

## 4 Discussion

MiR-143 and miR-145 act principally as tumor suppressors that are typically downregulated in several human cancers, including ovarian cancer. In this study, we explored the influence of two polymorphisms in the *miR-143/145* cluster on susceptibility to EOC and the clinical outcome of EOC patients. The results showed that the CC genotype of rs4705342 was associated with an increased risk of developing EOC, an advanced FIGO stage and poor prognosis of the EOC patients. Moreover, the carriers of the rs4705342 CC genotype exhibited a lower expression of miR-145 than those with the TT genotype. To our knowledge, this is the first study on the relationship between genetic variants of the *miR-143/145* cluster and EOC in Northern Chinese women.

Rs4705342 is an important polymorphism in the promoter of the *miR-143/145* cluster. Several studies have demonstrated that this variant could affect the transcriptional activity of *miR-143* and *miR-145*. Chu et al. (23) reported that compared with the rs4705342 C allele, the T allele could increase the protein-binding affinity and reduce the promoter activity. Sima et al. (24) found that intracranial aneurysm patients carrying rs4705342 CC/CT genotypes had higher levels of miR-143 than those carrying the TT genotype. In addition, Wei et al. (25) showed that the levels of miR-145 were significantly lower in the plasma from ischemic stroke patients carrying rs4705342 CC genotype. Similar to Wei’s results, the present study also found that the miR-145 levels were significantly decreased in the tumor tissues of EOC patients with the rs4705342 CC genotype compared with those with the TT genotype, but the miR-143 expression levels were not significantly different among the different genotypes of rs4705342. Our results further suggested that rs4705342 may be a functional polymorphism and affect the expression of miR-145 in EOC tissues. The association between rs4705342 and cancer risk has been examined extensively, but the results were controversial. The C allele of rs4705342 was found to play a protective role against the occurrence of papillary thyroid carcinoma (18). However, Kotarac et al. (19) reported that the rs4705342 C variant was associated with an increased risk of prostate cancer. In the present study, we also found that the frequencies of the rs4705342 CC genotype were significantly higher in the EOC patients than in the controls. Furthermore, the rs4705342 CC genotype was related to an advanced FIGO stage and poor prognosis of the EOC patients. Kim et al. (14) showed that low expression of miR-145 was significantly correlated with poor prognostic parameters, such as high FIGO stage and disease recurrence, and worse clinical survival. Based on these studies, we surmised that the rs4705342 CC genotype may significantly reduce the expression levels of miR-145 in EOC tissues, which may lead to the occurrence and progression of EOC, as well as shorter DFS and OS values in patients with EOC.

Rs353292 is a common genetic variant also in the promoter of the *miR-143/145* cluster. Yuan et al. (26) demonstrated that miR-143 levels were lower in colorectal tumor tissues carrying the rs353292 TT/CT genotype than in those carrying the rs353292 CC genotype, but no significant relationship was found between rs353292 and the expression of miR-145. Several studies have explored the association between this polymorphism and the risk of human diseases. The frequencies of rs353292 TT/CT genotype were found to be significantly higher in patients with colorectal cancer and chronic kidney disease (27, 28). However, the rs353292 polymorphism was not related to the risk of papillary thyroid carcinoma and conotruncal heart defects (18, 29). Similarly, our study also showed that rs353292 was not associated with susceptibility to EOC or the prognosis of EOC patients. Moreover, the expression levels of miR-143 and miR-145 were not significantly different in EOC tissues with different genotypes of rs353292. These findings suggested that rs353292 might have no effect on the risk of EOC or the clinical outcome of EOC patients in the Northern Chinese Han population.

There were some limitations in the present study. First, all recruited subjects were unrelated Han Chinese women from Shijiazhuang and the surrounding regions, and thus our results cannot be directly applicable to other ethnic or regional groups. Second, due to the lack of normal ovarian tissue specimens, we did not investigate the expression levels of miR-143 or miR-145 in the control group.

In conclusion, our study indicated that rs4705342 may be a functional genetic polymorphism that affects the expression of miR-145 in EOC tissues. The CC genotype of rs4705342 was associated with an increased risk of EOC and poor prognosis of EOC patients. Thus, rs4705342 may serve as a molecular marker for predicting the development of EOC and the clinical outcome of EOC patients. Of course, larger-scale and well-designed studies with multiethnic groups and longer follow-up times are required to verify our findings.

## Data availability statement

The original contributions presented in the study are included in the article/supplementary material. Further inquiries can be directed to the corresponding author.

## Ethics statement

The studies involving human participants were reviewed and approved by the Ethics Committee of the Fourth Hospital of Hebei Medical University. The patients/participants provided their written informed consent to participate in this study.

## Author contributions

TM: study design, data analysis, and manuscript revision. JZ: study design, manuscript writing, and data collection. WZu: manuscript writing, data analysis, and manuscript revision. YZ: data collection and data analysis. CH: manuscript writing and data analysis. WZh: manuscript revision. All authors contributed to the article and approved the submitted version.
